# Virus-Induced Genome Editing (VIGE): One Step Away from an Agricultural Revolution

**DOI:** 10.3390/ijms26104599

**Published:** 2025-05-11

**Authors:** Elena Mikhaylova

**Affiliations:** Institute of Biochemistry and Genetics, Ufa Federal Research Centre of the Russian Academy of Sciences, Ufa 450054, Russia; mikhele@list.ru

**Keywords:** viral vector, CRISPR/Cas, genome editing, transgene-free technologies, RNA-dependent RNA polymerase, virus-induced genome editing

## Abstract

There is currently a worldwide trend towards deregulating the use of genome-edited plants. Virus-induced genome editing (VIGE) is a novel technique that utilizes viral vectors to transiently deliver clustered regularly interspaced short palindromic repeat (CRISPR) components into plant cells. It potentially allows us to obtain transgene-free events in any plant species in a single generation without in vitro tissue culture. This technology has great potential for agriculture and is already being applied to more than 14 plant species using more than 20 viruses. The main limitations of VIGE include insufficient vector capacity, unstable expression of CRISPR-associated (Cas) protein, plant immune reaction, host specificity, and reduced viral activity in meristem. Various solutions to these problems have been proposed, such as fusion of mobile elements, RNAi suppressors, novel miniature Cas proteins, and seed-borne viruses, but the final goal has not yet been achieved. In this review, the mechanism underlying the ability of different classes of plant viruses to transiently edit genomes is explained. It not only focuses on the latest achievements in virus-induced editing of crops but also provides suggestions for improving the technology. This review may serve as a source of new ideas for those planning to develop new approaches in VIGE.

## 1. Importance of Transgene-Free Technologies in Agriculture

Stable agrobacterium-mediated transformation, first performed in the early 1980s, remains the most widely used technique in plant biotechnology. Transferred DNA (T-DNA), defined by conserved 25-base-pair imperfect repeats (left and right T-borders) is delivered from bacteria into the plant nucleus and integrated into the plant genome [1]. The presence of transgenes does not pose a problem for basic research as long as the resulting plant lines are not intended for commercial use. But persisting transgenes may pose regulatory, ethical, and safety concerns when it comes to commercialization [2]. In most countries, the cultivation of genetically modified organisms (GMOs) is prohibited, and there is a rather complicated procedure for bringing them to the market in countries where they can be legally grown. Only large companies can afford it, which results in the prevalence of traits such as herbicide tolerance and insect resistance.

Deregulation of transgene-free genome-edited plants in more than 20 countries opens up prospects for bringing them to global market [3]. It is believed that small base substitutions or indels induced by a CRISPR/Cas system without the integration of foreign DNA cannot pose any additional risks, compared to natural mutations. For example, the United States Department of Agriculture does not regulate genome-edited plants if they do not contain foreign DNA and if the modifications could have been achieved through conventional breeding. Similar decisions have been made in Argentina, Japan, Australia, and Brazil. Fast-track approvals for transgene-free genome-edited plants are supported in China and the UK. Deregulation efforts are ongoing in Europe [4].

As a result, many ambitious companies have emerged, aiming to commercialize gene-edited plants with novel traits, created using low-cost approaches. For example, Calyxt marketed transcription activator-like effector nuclease (TALEN)-edited high-oleic soybean, Sanatech Seed brought to the market high-gamma-aminobutyric acid (GABA) tomatoes developed using a CRISPR/Cas system, and GreenVenus LLC biotechnology commercialized non-browning avocados [5,6]. Universities and research organizations also own many CRISPR patents and patent applications [3].

The elimination of transgenes is one of the most time-consuming stages after stable plant transformation. There are some approaches, such as backcrossing, cross-pollination and RNA interference-based strategies, but they are labor-intensive and ineffective for vegetatively propagated crops. Direct delivery of ribonucleoprotein (RNP) consisting of Cas and guide RNA into plant cells has very low editing efficiency and many disadvantages, such as high cost and quick degradation of ribonucleoprotein complexes, requirement of protoplast culture techniques and the impossibility of using selective agents [7].

When stable genetic transformation is undesirable, viral vectors might be the best option. Plant viruses normally do not integrate into the plant genome because they replicate in the cytoplasm and lack integrase enzymes [8]. This makes them excellent tools for transgene-free genome editing.

Adenoviruses, lentiviruses, and adeno-associated viruses have become the most important genetic engineering and genome editing tools in animal science and medicine. In plant genome editing, viruses are used rather rarely and only by a small number of research groups. However, this area of research is rapidly evolving and expanding along with our knowledge about plant viral genomes.

## 2. Plant Viruses: Structure and Mechanism of Action

The International Committee on Taxonomy of Viruses develops, reviews, and updates the taxonomy and nomenclature of viruses. This organization currently recognizes around 1500 species of plant viruses (https://ictv.global/taxonomy (accessed on 15 March 2025)).

Most of them have single-stranded (ss) RNA genomes, which can be positive-sense (+), i.e., directly translated by the host’s ribosomes to produce viral proteins, or negative-sense (-), i.e., transcribed by a viral RNA-dependent RNA polymerase (RdRp). To infect plant cells, they unbind from their capsids and bind with movement proteins (MP), which are capable of increasing the size exclusion limit of plasmodesmata. Capsids of ss RNA viruses can be spherical, rod-shaped, or bullet-shaped [9].

The positive-sense group includes the *Virgaviridae*, *Potyviridae*, *Closteroviridae*, *Bromoviridae*, *Luteoviridae*, *Tombusviridae*, *Solemoviridae*, *Betaflexiviridae*, *Secoviridae*, and *Umbraviridae* families. The genomes of Potyviruses, Tymoviruses, Luteoviruses, Sobemoviruses, and Comoviruses are translated as one long polyprotein, which is then cleaved into functional proteins by proteases. Tobamoviruses, Closteroviruses, and Tombusviruses have multiple ORFs in the genome and a subgenomic RNA carrying its incomplete copies, which allow structural proteins to be made separately and flexibly, overcoming translation barriers [10].

Bunyaviruses, in particular the genus Tospovirus, are the only known group of (-) ssRNA plant viruses [11]. A lipid envelope derived from the host membranes makes them flexible and irregularly shaped. They carry pre-made RdRp inside the viral particles. Their genome is divided into three segments: L (large, encoding RdRp), M (medium, encoding two glycoproteins forming the viral envelope, which are essential for transmission by thrips), and S (small, encoding nucleocapsid and movement proteins, as well as suppressors of RNA silencing (VSRs)).

Double-stranded RNA (dsRNA) viruses, including the *Partitiviridae*, *Reoviridae* and *Chrysoviridae* families, are less common and are not usually used in biotechnology or plant genetic engineering [12]. The negative-sense strand of their genome is transcribed by viral RdRp into (+) RNA inside the capsid to avoid host immune detection. The newly synthesized RNA exits the capsid to be translated by the plant ribosomes. It also acts as a template for RdRp to synthesize complementary negative-sense RNA strands, resulting in the formation of new dsRNA genomes, which are further incorporated into new viral capsids.

Single-stranded DNA (ssDNA) viruses are represented by the *Geminiviridae* and *Nanoviridae* families [13]. Geminiviruses have some of the smallest genomes among plant viruses (2.5–3.0 kb). After the virus enters the plant nucleus, the host’s DNA polymerase synthesizes a complementary DNA strand. To avoid degradation by nucleases and RNAi mechanisms, dsDNA associates with host histones and form minichromosomes. Then, it serves as the template for mRNA transcription of viral proteins by the host RNA polymerase II and viral replication via a rolling-circle mechanism, initiated by the replication-associated protein (Rep) [14]. Most of ssDNA viruses are monopartite, but some, such as bean golden mosaic virus and tomato mottle virus, consist of two separate DNA molecules. The capsids of Geminiviruses have twinned icosahedral symmetry, giving them a unique hourglass shape.

Double-stranded DNA (dsDNA) plant viruses, represented by a single family, *Caulimoviridae*, are relatively rare [13]. Despite this, the most widely used promoter in plant biotechnology, CaMV 35S, originates from Cauliflower mosaic virus. The dsDNA virus releases its dsDNA, which is around 7.2 to 8.0 kb in size, in the nucleus, where accidental integration events can occur due to errors in plant DNA repair mechanisms and recombination between viral and host DNA. dsDNA viruses have spherical or rod-like virions and can move from cell to cell using MP.

Viral replication requires a large amount of energy and resources from the host cell, which affects many metabolic processes. Infection slows down photosynthesis and boosts respiration, which leads to accumulation of reactive oxygen species (ROS), reduction in chlorophyll content, and hormonal imbalance. Viruses can cause visual symptoms, such as mosaic patterns, leaf curling, chlorosis, and necrosis. Mosaic phenotypes arise due to uneven accumulation of high viral loads in plant tissues, where healthy and infected tissues coexist. Viruses can also significantly impact growth and yield. Dwarf viruses interfere with normal cell division and expansion and slow plant growth [15]. Since plants have rigid cell walls, viruses can only enter through wounds caused by insects—such as aphids and whiteflies—or nematodes or during physical contact, through contaminated tools or wind-blown plant debris. The wound disrupts the plant’s cell wall and plasma membrane, which normally act as barriers to infection. The virus particles spread either by adhering to receptors in the insect’s mouthparts or by being ingested. Binding is utilized by the capsid protein (CP) or specific proteins such as multifunctional helper component proteinase (HC-Pro) in Potyviruses [16]. In response to infection, plants employ an RNA silencing system (RNAi), which detects and eliminates transcripts from excessively expressed genes using small interfering RNAs (siRNAs) that target and degrade viral genomes. Host RdRp molecules are crucial in this process, leading to the amplification of the silencing signal by converting ssRNA into long dsRNA. For example, RDR1 is involved in the production of 21 nt long virus-activated siRNA (vasiRNA), produced as a result of the viral RNA cleavage by Dicer-like enzymes. vasiRNAs guide the Argonaute protein complex to the complementary viral RNA molecules to degrade the viral RNA or inhibit its translation, effectively silencing replication of the virus and production of new viral particles [17].

To avoid RNAi, many plant viruses have VSRs to inhibit the silencing pathway, allowing safe replication. For example, HC-Pro binds siRNAs, preventing them from being incorporated into the Argonaute protein complex, which is required for RNA-induced silencing complex activity [18].

Some viruses are passed to new plants through seeds or pollen, but it is generally less common than transmission by insects or mechanical means. Seed transmission of plant viruses happens predominantly in Caulimoviruses and Begomoviruses. They spread systemically through the phloem from infected tissues to flowers, where they can infect the embryo, ovule, or pollen before seed coat formation. This allows viruses to be directly transmitted to the next generation, ensuring infection in newly germinated plants. Some plants develop genetic resistance to seed transmission [19,20,21].

Deep knowledge of plant virus structure and functioning has opened up possibilities for their use as molecular tools in biotechnology, agriculture, and medicine.

## 3. Plant Viruses as Vectors

The idea to use plant viruses as vectors came up in the 1970s, Geminiviruses and Caulimoviruses being the first subjects of genetic manipulation [12]. This technology, based on the natural ability of viruses to deliver genetic material into cells, was initially applied for genome silencing (VIGS) and transient protein production [22]. Viral vectors are usually delivered to plant tissues via agroinfiltration with *Agrobacterium tumefaciens* [23]. Within plant cells, the contents of T-DNA can be assembled and expressed at a very high level without integration into the genome. Depending on the number of nucleocapsids, it can be delivered as a mixed suspension of bacteria cultures, carrying different plasmids. For example, cowpea mosaic virus (CPMV) has two RNA components, and tomato spotted wilt virus (TSWV) has three RNA components [24,25].

*Nicotiana benthamiana* has become the most valuable model plant in this area of research, because it is hypersusceptible to viruses, particularly positive-sense RNA viruses like Tobamoviruses, Potyviruses, and Tombusviruses. This is probably due to the lack of an active salicylic acid- and virus-inducible RdRP caused by a 72 nt insert in the *NtRdRP1* gene, which is necessary for efficient RNA silencing [26].

VIGS works by introducing a virus carrying a fragment of the plant’s own gene into the plant, triggering the natural RNAi antiviral defense system. Recognition of this fragment as foreign material results in gene knockdown, allowing us to study its role and functions [27]. The protein production mechanism is based on the ability of viral promoters to drive high transcription rates, leading to increased levels of mRNA, which is further processed by plant ribosomes. However, plant-based production systems have limitations, including the plant’s RNAi defense system against highly expressed genes, affecting the yield stability of desired proteins [28].

VIGE is a genome editing technique that uses engineered plant viruses to deliver CRISPR/Cas components into plant cells. Usually, viral genome is encoded inside the T-DNA borders of a binary vector, and the virus is assembled upon delivery to the plant cell. Due to the high replication rate, CRISPR/Cas components are expressed at a high level. Unlike traditional methods that require tissue culture and transformation, VIGE enables in vivo genome editing by systemically spreading editing reagents throughout the plant, potentially allowing for DNA-free, fast, and heritable edits without a regeneration step. Differences between VIGE and previous approaches are presented in Table 1.

**Figure 1:**
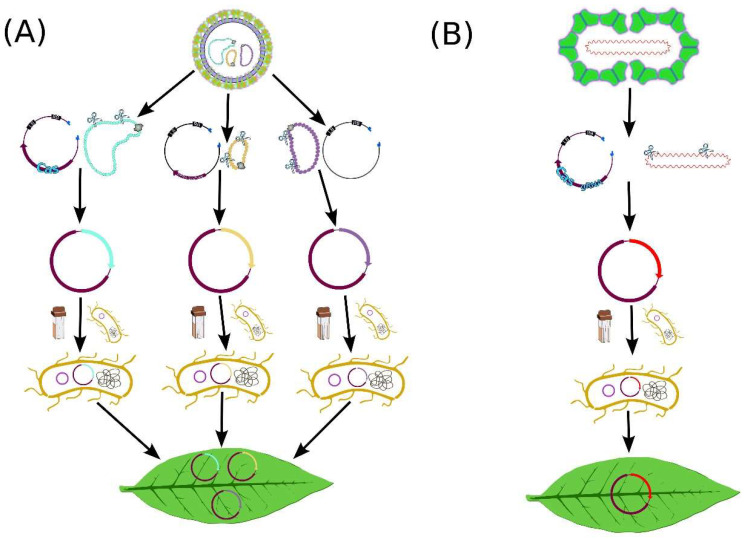
A scheme of agroinfiltration with (**A**) tripartite and (**B**) monopartite viral vectors. In brief, parts of the viral genome are introduced inside the T-DNA borders of one or several plasmids along with the *Cas* gene and gRNA cassette. The resulting vectors are cloned into *A. tumefaciens* via electroporation. One or several agrobacterium clones are used for agroinfiltration of plant leaves.

The most widely used method of virus delivery is agroinfiltration with *A. tumefaciens* (Figure 1). The modified virus capable of replication is cloned into a T-DNA region of a binary plasmid and introduced into Agrobacterium. Bacterial cultures are then resuspended in an infiltration medium and injected into plant leaves using a needleless syringe or vacuum infiltration. Transfer of the T-DNA into plant cells in the infiltrated tissues induces transient viral replication and expression of viral proteins [29].

The efficiency of this approach may be reduced because *A. tumefaciens* and viruses compete for the same cellular resources. Moreover, bacterial infection activates biotic stress response, which can interfere with viral replication, assembly, and the overall success of these virus-based techniques. Plants can destroy the infected cells, activate the salicylic acid pathway and pathogenesis-related proteins. Therefore, low-virulence strains such as EHA101, LBA4404, and GV3101 are most often used [30]. A. tumefaciens is also considered to have limited success in monocots (such as wheat, rice, or maize) due to their thicker cell wall and lack of receptors for Agrobacterium on the cell surface. Efficient transformation of these species requires explants with a high proportion of actively dividing cells, namely, immature embryos. Moreover, monocots do not secrete acetosyringone in wounded areas, unlike dicotyledonous plants [31]. The involvement of Agrobacterium can also result in stable integration of viral constructs or their parts, because T-DNA is specifically designed to integrate into the plant’s genomic DNA [32]. Viruses can also be introduced directly in the form of viral cDNA, PCR products or viral vector constructs by mechanical inoculation methods, such as rubbing with an abrasive like carborundum powder with cotton swabs onto the leaf surface. The abrasive material gently breaks the plant’s cuticle and cell walls, creating small openings through which the virus can enter. If it retains the ability for replication, systemic viral infection occurs, and the virus spreads throughout the plant from the initial infection site. Healthy plants can be further infected by grinding up tissues of systemically infected plants in inoculation buffer and rubbing them on the leaves of a recipient plant using the abrasive. The efficiency of this approach is usually lower compared to Agrobacterium-mediated transformation or biolistics [33]. A hand-held particle bombardment device allows us to inoculate intact plants with particles coated with viral genetic material or whole virions from infected tissues. This approach is frequently used with plant species that are not susceptible to Agrobacterium, such as apple, pear, soybean, and cassava. It is also more efficient and accurate in wounding plant cells than mechanical inoculation [34].

Among the most often used viral backbones are those of the RNA viruses tobacco mosaic virus (TMV), potato virus X (PVX), cowpea mosaic virus (CPMV), and tobacco rattle virus (TRV), as well as the DNA Geminivirus bean yellow dwarf virus. They have a broad host range, minimal effects on plant growth, and a high replication rate [29]. However, a large number of more specific viral vectors have been developed, which are presented in Table 2.

As can be seen from the table, a fairly large number of plant viruses have been used as vectors for VIGS and protein expression, and many of them have already found application in VIGE. Among them, agriculturally important ssRNA (+) and ssDNA viruses are the most widely employed. Viruses with high pathogenicity, high risks of escape into the environment, complex transmission mechanisms, large genomes, or difficulty in genetic manipulation are usually not considered suitable vectors. For example, tobacco necrosis virus (TNV) has a small genome of just 1.4 kb, has a limited host range, and is transmitted through fungi. Raspberry ringspot virus (RpRSV), lettuce big-vein virus (LBVV), Arabis mosaic virus (ArMV), and grapevine fanleaf virus (GFLV) also have never been used as vectors. Application of the virus may also be limited by the lack of research, especially if the infections they cause are asymptomatic. There are still a huge number of viruses that are poorly studied or not even discovered yet, especially among the viruses of wild plants that are not used in agriculture. Over the past few decades, advancements in next-generation sequencing (NGS) have dramatically increased the ability to detect previously uncharacterized viruses in plants [100,101]. Expanding our knowledge of plant viruses may lead to the discovery of the perfect vector.

## 4. Recent Advances in VIGE

Viral delivery can be successfully used for CRISPR/Cas genome editing, as it requires only the expression of Cas protein and one or several guide RNAs [7]. Although VIGE technology has only been adopted for a few years, promising results have already been achieved. At least 22 different viruses have been adapted to deliver genome editing tools (Table 2). However, it cannot yet be stated that a suitable vector that will revolutionize agriculture through genome editing has been developed. It is difficult to maintain a balance between optimal conditions for CRISPR/Cas machinery, replication and spread of the virus, and plant growth and development.

In earlier studies, viruses were used only to deliver gRNAs to transgenic plants that stably expressed Cas protein [102]. Increasing the maximum size of heterologous protein has not really been a problem for VIGS and protein expression systems. But frequently used plant viral vectors, such as TRV, appeared to have too small cargo capacity to carry Cas9. It inspired researchers to further develop the technology towards bypassing stable transformation and tissue culture by transiently expressing the entire CRISPR/Cas machinery. Nevertheless, the original method is still used to study various factors that can improve editing efficiency [56,67].

**Figure 2:**
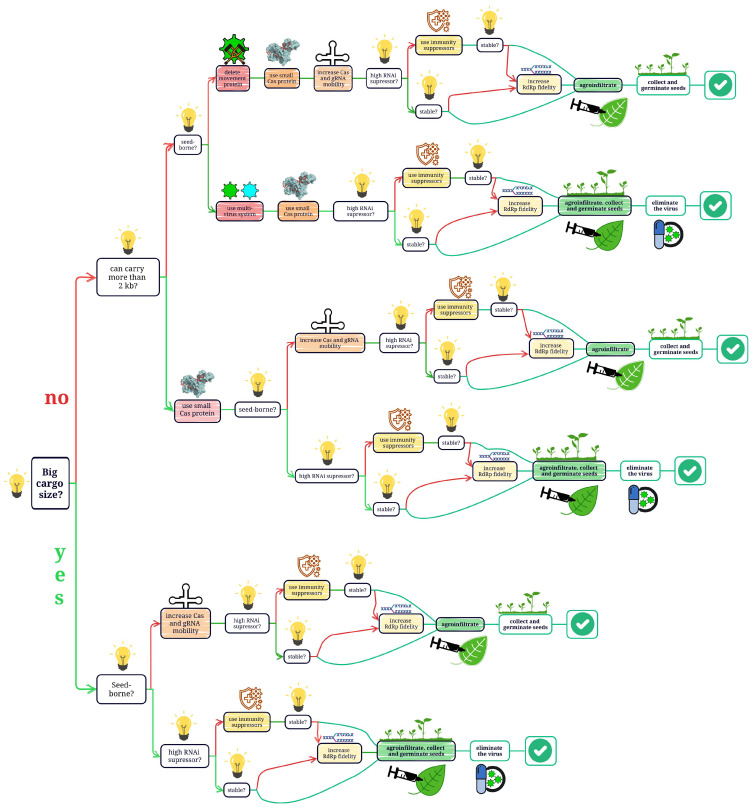
Decision tree algorithm illustrating the possibilities of improving VIGE. Depending on the characteristics of the virus, several approaches (application of small Cas protein, mobile elements, immunity suppressors, multivirus system, removal of movement protein, and improvement of RdRp) can be used for transgene-free, tissue-culture-free genome editing. The red arrows represent negative answers, and the green arrows represent positive answers.

Currently, scientists use unarmed or deconstructed viruses, free from unwanted functions, such as transmission with insects. There are several key issues that need to be addressed before VIGE can be widely adopted, and the global scientific community is still searching for solutions. These problems are complex, depending on one another. For example, most plant viruses are not seed-borne, and those transmitted through seeds may not have enough cargo capacity. Removal of viral proteins involved in transmission in order to increase cargo capacity comes at the cost of mobility and requires tissue culture, which is problematic for crops. The vast majority of experiments were carried out only on a model plant, *N. benthamiana*, and may not work well for other species. The transgene-free approach does not allow for selection using common agents such as antibiotics, herbicides, and fluorescent proteins, making identification of editing events a labor- and resource-intensive process and tempting researchers to target only the *Phytoene desaturase* (*PDS*) gene. A high expression level of CRISPR/Cas machinery is required for successful editing, but at the same time, it triggers plant immune response. Research is actively being conducted on each of the current problems of VIGE, and several solutions have been proposed [102]. The algorithm for applying these solutions is presented in Figure 2.

### 4.1. Increasing Cargo Capacity

The cargo capacity of viral vectors is important for transgene-free genome editing. Inserting large or multiple genes can result in genome size limitations being exceeded, causing the viral vector to become unstable or non-viable. A vector (or several vectors assembling into a multipartite virus) should express both Cas protein and gRNAs to avoid the development of transgenic plants expressing the nuclease. In some cases, donor DNA for knock-in of exogenous genes may also be required [35]. CCP, MP, VSRs, and non-coding regulatory sequences can be sacrificed to increase cargo capacity. The shape of the virus limits maximal insertion size. Spherical viruses have fixed dimension; however, filamentous viruses are extendable without any physical limits [12]. But real capacity is experimentally confirmed only for a small number of viral vectors.

It is known that Rhabdoviruses are capable of expressing up to 6 kb of foreign DNA. The entire CRISPR/Cas machinery was successfully delivered using BYSMV- and SYNV- derived vectors [80,81]. But these viruses have a narrow host range and do not infect the germline; therefore, they cannot be used for many agricultural crops.

Potexviruses can carry large cargoes due to their flexible structure. FoMV, which has a wide host range, may be considered one of the most promising VIGE vectors [69]. PVX also proved capable of delivering Cas9, but it predominantly infects *Solanaceae* plants, which imposes restrictions on the use of this virus [65]. Tripartite Bunyaviruses are also showing promise despite being only recently applied as vectors. Due to their relatively large genomes, they can carry not only a CRISPR/Cas system but also reporter genes, such as GFP, and retain infectivity [25]. Benyviruses have a multipartite genome and many proteins that can be replaced without affecting the functionality of the virus. At least four proteins can be expressed by BNYVV-based vectors, including those with lengths of up to 880 amino acids [76]. Potexviruses, Bunyavirususes, and Benyviruses also are not seed-transmitted.

Viral vectors derived from Bromoviruses and Geminiviruses with icosahedral virions usually cannot contain an insertion longer than 300 bp. But since only Rep protein and a specific intergenic region are required for the replication of Geminiviruses, they can be used as Rep-dependent protein expression platforms after removal of the CP and MP. This lifts constraints on the size of the genome and allows the delivery of big cargoes at the cost of virus mobility [103]. However, the removal of components that provide cell-to-cell movement is undesirable in genome editing, since it predetermines in vitro culture stages [22].

### 4.2. Increasing Stability of Viral Vectors

There is a negative correlation between insertion size and vector stability [104]. Therefore, it is not enough to just increase the vector capacity; it is also important to ensure the stability of the insert. Foreign inserts, especially those containing duplicated elements, are often not maintained stably over time because of recombination events. This can result in the deletion or rearrangement of the inserted transgene, leading to diminished or lost expression. Foreign sequences might also disrupt the secondary and tertiary structure of the virus, which is required for its expression [81]. Moreover, RdRps of RNA viruses lack proofreading capabilities, leading to high mutation rates during viral replication. This problem is difficult to overcome because the instability of viruses is an advantage for their evolution [105]. Exchange of genetic material between different viral genomes and spontaneous mutations contribute significantly to genetic diversity, altered pathogenicity, adaptability, and host range of plant viruses.

Some viruses are also more stable than others. For example, cucumber mosaic virus produces predominantly defective RNA during infection [87]. On the other hand, SYNV demonstrates low homologous recombination rates. Along with extendable bullet-shaped structure, it accounts for the large cargo capacity of SYNV. Therefore, careful selection of the virus, the host, and the insert is key to successful genome editing.

As one of the possible solutions to increase stability, the fidelity of RdRp can be increased. Specific mutations to the active site, contributing to a less active open conformation, lead to slower nucleotide incorporation and increased selectivity [106]. Introns and high GC content also contribute to better stability in viral vectors [81].

### 4.3. Decreasing the Size of Cas Protein

The search for new smaller Cas proteins may be considered as an alternative strategy for increasing the cargo capacity. Genes for very small nucleases might fit into the seed-transmitted viral vector without affecting its ability to move. This problem is primarily important for the editing of mammalian cells, so the search for new proteins is being intensively carried out. Currently, spCas9 derived from *Streptococcus pyogenes* and Cpf1 (Cas12a, from *Francisella novicida*) are the most widely used. They are considered rather big (1307–1368 aa, about 4.1 kb in length), compared to novel miniature CasΦ (Cas12j) and Cas12f1, which are less than half their size [47,107,108,109,110]. Cas12f, also known as Cas14, is probably the smallest Cas protein, which is sourced from archaea and consists of only about 400 to 700 aa [111].

New Cas proteins have been discovered regularly in recent years. They vary in size, typically ranging from 200 to 1400 amino acids. The Cas Protein Data Bank currently contains 287 known and 257,745 putative proteins [112].

### 4.4. Increasing Mobility of CRISPR/Cas System

Delivery of the CRISPR/Cas machinery to generative organs is essential in transgene-free VIGE in agricultural crops. Tissue culture has been successfully used in *N. benthamiana* for viral vectors that are not seed-transmittable or incapable of movement, but for many plant species and varieties it is problematic [64,81]. Regeneration protocols for most non-model plants are very complicated or have not been developed at all.

Plants are capable of long-distance transport of RNAs via phloem and plasmodesmata, which provide cytoplasmic connection between cells through microscopic channels. The secondary structure of these RNAs is recognized by RNA-binding proteins and facilitates the selective movement of RNAs by the transport machinery [102,113]. Fusion of such mobile elements to the 3′ end of gRNAs has been successfully used for plants stably expressing Cas protein. Flowering Locus T (FT) mRNA is a mobile RNA capable of long-distance movement within plants due to the presence of a specific cis-acting element, enabling its systemic trafficking to the reproductive organs. It serves as a key regulator of the transition from vegetative growth to flowering, enabling plants to synchronize flowering with favorable environmental conditions. An FT sequence can be either indigenous or derived from *A. thaliana*. gRNA-FT fusions allowed to achieve virus-free edited progeny in arabidopsis, tomato, and tobacco with the CLCrV, TRV, and PVX vectors [51,55,61,64]. However, experiments with FoMV in tobacco and maize, CLCrV in cotton, and BSMV in wheat failed to improve the efficiency of heritable gene editing [50,73,114].

tRNA-like sequences (TLSs) within mRNAs resemble the stem-bulge–stem-loop structure of transfer RNAs and therefore can significantly enhance long-distance transport through a plant’s vascular system [102,113,115]. Both FT and tRNA approaches have been used in tomato genome editing, demonstrating that TRV is superior to the PVX vector, with efficiencies as high as 62.1% and 65% [56]. gRNA-TLS fusion was also successfully applied to achieve heritable edits in *A. thaliana* and *N. benthamiana* with TRV viral vectors [61,116,117]. The frequency of heritable edits could increase by up to 100% depending on the target gene. With BSMV as well as FT mRNA, this approach failed [73].

For some reason, there have been no attempts to use mobile elements to ensure cell-to-cell movement of Cas transcripts, even though this is possible. Mobile Cas9 has recently been implemented in genome editing by grafting. Edited non-transgenic plants were found among the seed progeny of shoots grafted on the roots expressing mobile Cas9 and gRNAs [81]. However, the editing efficiency was only 0.6%, and only one-fifth of the edited plants had a homozygous mutation. So, the efficiency of using mobile nucleases in the grafting format is questionable, since their expression in the roots is insufficient. In addition, for many species of crop plants, the creation of transgenic roots and the grafting of shoots onto roots is problematic or impossible. It can be assumed that expression from viral vectors would significantly increase production of transcripts and editing efficiency. A significant number of mobile transcripts from different plant species contain TLS in the CDS or 3′-UTR, which indicates the possibility of using this sequence to ensure the mobility of CRISPR system elements in a large number of agricultural crops [115].

### 4.5. Decreasing Host Immune Response

Viral vectors can interrupt the normal biosynthetic processes in a cell, because they use the host’s transcriptional and translational machinery to express viral genes and replicate, which reduces the synthesis of the plant’s own proteins. Host defense mechanisms play an important role by recognizing, targeting, and degrading viral vectors, particularly those expressing foreign proteins. Plants generally utilize RNAi mechanisms for this purpose, such as post-transcriptional gene silencing [118].

This can be overcome with viral suppressors of post-transcriptional gene silencing, such as TEV helper component–proteinase. HC-Pro allowed an increased accumulation of GFP by up to ∼3% of total protein without viral symptoms [119]. VIGS can be used along with VIGE to knockdown genes of a plant’s antiviral RNA silencing system. Targeting of *N. benthamiana* RdRp 6 using an ALSV vector that proved efficient in VIGS improved TRSV-mediated gene editing by 0.8–13.2%. Modification of this TRSV vector to additionally encode the 16K protein from TRV, which acts as a VSR, contributed to more severe infection symptoms but did not significantly improve editing efficiency [57]. Viruses with naturally high RNAi suppressor activity, such as TBSV, can be used to deliver CRISPR/Cas components [99]. The P19 protein from this virus can also be cloned to another viral vectors, such as FoMV, to enhance levels of Cas protein in infected leaf tissues [69]. Multiple RNAi suppressors, including HC-Pro, p19, and gamma-B protein derived from BSMV, can be co-delivered separately along with VIGE vectors [25].

It is important to note that some plants have a stronger immune response, and some are more susceptible. Most of the above-listed research involves only *N. benthamiana*; however, other plant species may utilize other defense mechanisms, such as resistance genes and recognition receptors, which require other approaches.

### 4.6. Multivirus Vector Systems

Co-infection of a plant with more than one virus type may cause a synergistic effect, increasing the effectiveness of VIGE. Therefore, double and triple infections are therefore frequent in natural conditions [98]. During co-infection, certain viruses may complement each other’s functions, leading to a boost in their collective replication and movement within the plant. Changes in gene expression or metabolic pathways induced by one virus might create conditions that favor the replication or movement of the co-infecting virus. The combined action of RNAi suppressor proteins of different viruses can more effectively inhibit the plant’s immune responses [120]. By distributing the Cas protein and gRNA across two separate vectors, this system effectively bypasses the size limitations inherent to single-virus vectors. For example, delivery of sgRNAs in a PVX vector, combined with a TEV vector transiently expressing a Cas12a nuclease, allowed targeted mutagenesis in wild *N. benthamiana* with a high efficiency of 20% [97]. Double infection with TRSV vectors carrying Cas9 and TRV carrying sgRNA successfully introduced systemic mutations in tobacco [56,57].

A combination of FoMV constructs were used in genome editing of tobacco [69]. Three individual genetic constructs were used to deliver three particles of TSWV, which were subsequently assembled in the cell [25]. To date, double- and triple-virus approaches are not yet common in plant science. However, they are frequently used in animal models, suggesting that this method may be promising for VIGE.

### 4.7. Regulating the Temperature Conditions

Temperature significantly influences the replication and spread of plant viruses, impacting both the virus’s life cycle and the host plant’s defense mechanisms. Higher temperatures can increase membrane fluidity and cytoskeletal dynamics within plant cells, facilitating infection and the development of symptoms. Cooler temperatures have been associated with decreased viral replication rates and stress damage [121]. Therefore, the efficiency of VIGE can be regulated by changing temperature conditions.

TRV and PVX vectors demonstrated higher editing efficiency at 20 °C compared to 25 °C. Incorporating low-temperature conditions during VIGE at the initiation stage of tissue culture in tomato increased editing efficiency, resulting in mutation rates exceeding 70% in regenerated plants [56]. But the applicability of this method is questionable because Cas proteins have their own optimal temperature, which is as high as 32 °C for SpCas9 and 28–32 °C for Cas12a [7]. However, this approach might be used with hypercompact CasΦ-2 nuclease, which has a wide functional temperature range between 23 °C and 28 °C [107].

### 4.8. Providing Seed Transmission

Plant meristems have smaller plasmodesmata and high levels of auxin, which restrict viral movement and replication, making plant generative organs virus-free [118]. Many viruses, such as Geminiviriuses and Closteroviruses, can penetrate this barrier. Approximately one-third of plant viruses, including Partitiviruses, dsDNA Caulimoviruses, and many +ssRNA virus families, can be transmitted through ova, pollen, or seeds, but usually with low efficiency. They do not necessarily infect the seed embryo but can be transmitted to seedlings by contamination of the seed coat [87]. The percentage of transmission is high in sweet potato leaf curl virus, soybean mosaic virus, and papaya meleira virus (>70%); however, these viruses are not yet used in VIGE. Seed transmission also depends on the host species, which can have different control mechanisms. Viruses utilize suppressors of RNAi to mediate vertical transmission [19].

TRV and BSMV, known for their ability to be transmitted through seeds, have been widely employed to deliver guide RNAs (gRNAs) into Cas9-expressing plants [55,73,74,75]. However, they are incapable of carrying Cas nuclease genes. Bipartite ssRNA (+) virus TRSV allowed tissue-culture-free delivery of both Cas9 and sgRNA, with a 5.6% seed transmission rate in tobacco; however, its relative ALSV was not detected in the seeds [57].

It is of great interest to identify other viruses that would allow the delivery of the full CRISPR/Cas machinery. However, the large size of the nuclease may prevent the viral vector from passing through the smaller meristem plasmodesmata. Moreover, seed-transmitted viral vectors will require further elimination of the virus in order to produce healthy gene-edited plant lines.

### 4.9. Elimination of Viruses

For seed-borne viruses and symptom-causing viruses, removal after successful editing is of great importance. This is a challenging task because no antiviral drugs are available against plant viruses. It has been shown that TSWV antiviral treatment with ribavirin during tissue culture allowed the recovery of 100% virus-free plants. The efficiencies of favipiravir and remdesivir were much lower (66.7% and 15.6%). Plants treated with remdesivir displayed infection symptoms and set no or substantially fewer seeds [25]. There are data on the successful use of other drugs to eliminate plant viruses [118]. Virazole and amixin were active against Tobamoviruses and the complex of rose mosaic viruses but had a pronounced toxic effect. 2thiouracil and 5-azadihydrouracil inhibited the reproduction of TMV, PVX, PVY, and CMV. Ningnanmycin and cytosinpeptidemycin can induce systemic resistance to TMV. Vanisulfane is active against CMV, PVY, and pepper mild mottle virus (PMMoV).

There is another approach based on RNAi technology, which allows the removal of RNA virus by the host’s native defense system, activated during a certain stage of a life cycle, by introducing a target site for host miRNA to the vector. The expression of tobacco microRNA398 is induced during shoot regeneration. The introduction of its target site to the vector allowed the elimination of the vector after edited shoots were regenerated from the infected leaves [78]. It is unlikely that this approach will be widely adopted in the near future due to the lack of knowledge about the targets and expression profiles of miRNAs in non-model plant species.

### 4.10. Application of VIGE in Crops

*N. benthamiana* is notably susceptible to a wide range of plant viruses due to its genetic deficiencies. Therefore, the obtained results may not be applicable to agricultural crops. There are a small number of plant species that have been successfully edited using viral vectors; however, these comprise the world’s most important crops.

Tomato is the most popular object of genome editing after *N. benthamiana* and *A. thaliana*. This is possible mainly due to the well-developed protocols for in vitro transformation of this species [35,55,56,58,78,122,123]. Other *Solanaceae* crops can be edited using similar tools, as they can share common viral infections with tobacco [55]. Genome editing has been performed on potato [36,55], eggplant [55], and pepper [54]. Successful VIGE has been reported in cassava with CsCMV vectors [42] and in cotton with BeYDV vectors [37]. ALSV has been used for the editing of soybean [84].

Different viruses are efficient in monocots. For example, FoMV was successfully used in sorghum, maize, and foxtail millet [67,68]. Barley, wheat, and maize were edited using BSMV vectors [74,75]. WDV replicons were used for VIGE in rice and wheat [38,39]. It is well known that monocots, especially barley, are harder to manipulate compared to dicots. They require specialized protocols due to the narrower range of efficient Agrobacterium strains, lack of pluripotent cells in mature tissues, and tissue culture recalcitrance in most of the genotypes. As a result, protocols for monocots rely on highly specific explants (immature embryos, microspores, or inflorescences) rather than leaves or stems, which may take a year or more to produce edited seeds, slowing research and breeding. VIGE, which allows us to bypass tissue culture, is a promising technique for monocots. Viral vectors can be propagated in a highly susceptible species such as *N. benthamiana* and then introduced to monocotyledon plants as assembled infectious viral particles. Viruses like BSMV naturally infect a wide range of monocot species and cultivars.

VIGE has never been applied in minor crops such as amaranth, banana, papaya, cucumber, pumpkin, buckwheat, quinoa, cabbage, oilseed rape, and many others. Minor crops play crucial roles in enhancing dietary diversity, ensuring food security, and supporting sustainable farming practices. They may also form the basis of the diet or trade of certain countries and regions. Since large corporations have little interest in these plants, the scientific community has the opportunity to take the initiative in this area and create the first transgene-free edited varieties using VIGE.

### 4.11. Targeting Economically Valuable Traits

Despite the absence of stably integrated selective markers in VIGE technology and the preference of the *PDS* gene as a target, several other genes have already been edited [102]. Among them are *GASR7* and *GW2*, associated with grain size; *tms5,* which controls thermo-sensitive genic male sterility; *HKT1,* conferring salt tolerance; *FWA,* which controls flowering, and others. There is a large number of quality-enhancing pathogen and abiotic stress resistance genes [6], *acetolactate synthase* gene [36,103], and specific genes responsible for various phenotypic traits such as anthocyanin accumulation [35,124] and trichome development [125] that can be considered suitable targets for VIGE.

## 5. Conclusions

Transgene-free technologies represent an exciting frontier in modern agriculture with significant potential for commercialization. It is necessary to move on from conventional stable transformation and develop technologies for transient expression without traditional selection markers, which primarily lead to heritable editing. Researchers have long failed in delivering the entire CRISPR–Cas machinery in a single virus-derived vector and ensuring its transmission to the meristem. However, in recent years, a large number of approaches have been developed to achieve this goal. These innovative techniques include the fusion of mobile elements, RNAi suppressors, novel miniature Cas proteins, and seed-borne viruses, which should be combined in order to achieve the desired effect.

One of the most successful heritable and tissue-culture-free gene editing methods utilizes a TRSV-TRV-ALSV triple vector system carrying Cas9 and FT:gRNAs along with a RNAi suppressor [57]. However, this approach has so far only been applied to the *N. benthamiana PDS* gene.

For most researchers working in the field of CRISPR/Cas, the choice between the simplicity of selection processes and transgene-free technologies, between transient and stable expression, and between in vitro culture and agroinfiltration has not yet been made. Before VIGE can be widely adopted, several key issues need to be addressed.

## Figures and Tables

**Table 1:** Comparison of plant genome manipulation methods.

	VIGS	Transient Protein Production	Agrobacterium-Mediated Genome Editing	RNP-Mediated Genome Editing	VIGE
Use of virus	Yes	Yes	No	No	Yes
Goal	Silence gene expression	Short-term production of recombinant proteins	Easy selection of editing events	DNA-free genome editing	Transgene-free, in planta genome editing
Mechanism	A fragment of the target gene, delivered by viral vector, triggers the immune response	Viral vectors ensure high expression levels of recombinant proteins	The same as transgenesis	Delivery of RNPs by protoplast electroporation or particle bombardment	Transient expression from viral vector
Genome Integration	No	No	Yes	No	No
Tissue culture	No	No	Yes (except Arabidopsis)	Yes	Not tissue-culture-free yet
Heritability	No	No	Yes	Yes	Potentially yes
Key Applications	Functional genomics, pathway validation	Vaccine/antibody production	Genome editing in varieties susceptible to agrobacterium-mediated transformation	Genome editing in varieties with well-developed protoplast isolation and regeneration protocols	Genome editing of varieties infected by certain viruses

**Table 2:** Plant viruses used as vectors.

Genus	Type and Shape	Genome	Transmission	Virus	Hosts	VIGE	References
Geminivirus, Mastrevirus	Twinned icosahedral circular monopartite	ssDNA, 2.5–3.0 kb	Leafhoppers	Bean yellow dwarf virus (BeYDV);	French bean (*Phaseolus vulgaris*), tobacco, tomato, potato, *Datura stramonium*, *Arabidopsis thaliana*	+	[35,36,37]
				Wheat dwarf virus (WDV)	Wheat, barley, oats	+	[38,39]
				Maize streak virus (MSV)	Maize, other grasses	-	[40]
Geminivirus, Begomovirus	Twinned icosahedral circular bipartite	ssDNA, each 2.6–2.8 kb	Whiteflies	Cabbage leaf curl virus (CaLCuV)	Cabbage, other crucifers	+	[41]
				African cassava mosaic virus (ACMV)	Euphorbiaceae family	+	[42]
				Tomato golden mosaic virus (TGMV)	Tomato, other solanaceous plants	-	[43,44]
				Pepper huasteco yellow vein virus (PHYVV)	Peppers, other solanaceous plants	-	[45]
				Tomato yellow leaf curl China virus (TYLCCNV)	Tomato, other solanaceous plants	-	[46,47]
				Honeysuckle yellow vein virus (HYVV)	Tomato, other solanaceous plants	-	[48]
				Chilli leaf curl viruses (ChLCV)	Chili peppers, other solanaceous plants	-	[49]
				Cotton leaf crumple virus (CLCrV)	Cotton, other malvaceous plants	+	[50,51]
				Bhendi yellow vein mosaic virus (BYVMV)	Okra (bhendi), other malvaceous plants	-	[52]
	Monopartite	ssDNA, 2.6–2.8 kb	Whiteflies, seeds	Sweet potato leaf curl virus (SPLCV)	Ipomoea	+	[53]
Geminivirus, Curtovirus	Twinned icosahedral circular monopartite	ssDNA, ~3.0 kb	Leafhoppers	Beet mild curly top virus (BMCTV)	Beets, other chenopods	-	[48]
Bunyavirus, Tospovirus	Spherical or pleomorphic tripartite	ssRNA (-), parts: L (8.9 kb), M (4.8 kb), and S (2.9 kb)	Thrips, seeds (in some hosts)	Tomato spotted wilt virus (TSWV)	Over 1090 dicotyledonous and monocotyledonous species	+	[25,54]
Tobravirus, Virgaviridae	Rod-shaped bipartite	ssRNA (+); RNA1, ~6.8 kb; RNA2, ~1.9–4.3 kb	Nematodes, soil, seeds	Tobacco rattle virus (TRV)	Tobacco, potato, over 400 species from 50 families	+	[55,56,57,58]
		ssRNA (+); RNA1, ~7.1 kb; RNA2, ~3.5 kb		Pea early-browning virus (PEBV)	30 legume species, N. benthamiana, A. thaliana	+	[59,60,61]
		ssRNA (+); RNA1, ~6.8 kb; RNA2, ~1.7 kb		Pepper ringspot virus (PRSV)	Pepper, tomato, artichoke, potato	-	[62,63]
Potexvirus, Alphaflexiviridae	Flexuous rod monopartite	ssRNA (+), ~6.4 kb	Mechanical	Potato virus X (PVX)	Mostly limited to Solanaceae	+	[64,65]
				Foxtail mosaic virus (FoMV)	56 monocot species and 35 dicot species	+	[66,67,68,69]
				Cassava common mosaic virus (CsCMV)	Cassava	+	[42]
				Pepino mosaic virus (PepMV)	Mostly limited to Solanaceae	-	[70]
Tymovirus	Monopartite icosahedral	ssRNA (+), ~6.3 kb	Beetles, seeds	Turnip yellow mosaic virus (TYMV)	*Brassicaeae*	-	[71,72]
Hordeivirus, Virgaviridae	Rod-shaped tripartite	ssRNA (+), total ~12.7 kb	Mechanical, seeds, soil	Barley stripe mosaic virus (BSMV)	Barley, wheat, other gramineous plants	+	[73,74,75]
Benyvirus, Flexiviridae	Filamentous multipartite	ssRNA (+), total ~15–16 kb	*Polymyxa* *betae* (soil)	Beet necrotic yellow vein virus (BNYVV)	*Chenopodiaceae*, *Amaranthaceae, Caryophyllaceae*	+	[76]
Tobamovirus, Virgaviridae	Rod-shaped monopartite	ssRNA (+), 6.4 kb	Mechanical	Tomato mosaic virus (ToMV)	Over 200 species	+	[77,78]
				Tobacco mosaic virus (TMV)	Over 125 species	+	[79]
Cytorhabdovirus, Rhabdoviridae	Bullet-shaped monopartite	ssRNA (-), 12.7 kb	Planthoppers	Barley yellow striate mosaic virus (BYSMV)	Poaceae	+	[80]
Nucleorhabdovirus, Rhabdoviridae	Bullet-shaped monopartite	ssRNA (-), ~13 kb	Aphids	Sonchus yellow net virus (SYNV)	*Compositae*, *Solanaceae*, and *Chenopodiaceae* families	+	[81]
		ssRNA (-), ~14 kb	Leafhoppers, mechanical	Eggplant mottled dwarf virus (EMDV)	Wide host range (*Solanaceae*, *Cucurbitaceae*, *Chenopodiaceae*, *Amaranthaceae*, *Malvaceae*, *Hydrangeaceae*, *Caprifoliaceae*, *Geraniaceae*, *Pittosporaceae*)	+	[82]
Comovirus, Secoviridae	Icosahedral bipartite	ssRNA (+); RNA1, ~5.8 kb; RNA2, ~3.5 kb	Leaf-feeding beetles, mechanical, seeds	Cowpea mosaic virus (CPMV)	*Fabaceae*	-	[24,83]
		ssRNA (+); RNA1, ~6.8 kb; RNA2, ~3.3 kb	Nematodes, seeds	Apple latent spherical virus (ALSV)	*Caryophyllaceae*, *Chenopodiaceae*, *Cryptomeria*, *Fabaceae*, *Cucurbitaceae*, *Gentianaceae*, *Pinus*, *Rosaceae*, *Rutaceae*, *Solanaceae*, *Arabidopsis*	+	[57,84,85]
Nepovirus, Secoviridae	Icosahedral bipartite	ssRNA (+), 4.8 and 7.2 kb	Nematodes, thrips, seeds, mechanical	Tobacco ringspot virus (TRSV)	Broad host range (*Solanaceae*, *Fabaceae*, *Cucurbitaceae*, woody plants)	+	[57,86]
Caulimovirus, Caulimoviridae	Icosahedral monopartite	dsDNA, 8 kb	Aphids, seeds	Cauliflower mosaic virus (CaMV)	*Brassicaceae*	-	[87]
Caulimovirus, Tungrovirus	Bacilliform circular monopartite	dsDNA, ~8.3 kb	Leafhoppers	Rice tungro bacilliform virus (RTBV)	Rice	-	[88,89]
Cucumovirus, Bromoviridae	Icosahedral tripartite	ssRNA (+), total ~8.6 kb	Aphids, seeds	Cucumber mosaic virus (CMV)	Over 1300 species	-	[90,91]
Bromovirus, Bromoviridae	Icosahedral tripartite	ssRNA (+), total ~8.6 kb	Mechanical, spotted cucumber, beetles, nematodes	Brome mosaic virus (BMV)	Monocotyledonous cereal crops	-	[92,93]
Potyvirus, Tritimovirus	Filamentous monopartite	ssRNA (+), ~9.4 kb	Mites, mechanical, low rates of seed transmission	Wheat streak mosaic virus (WSMV)	Poaceae	-	[94]
Potyvirus	Filamentous monopartite	ssRNA (+), ~9.8 kb	Aphids, mechanical	Plum pox virus (PPV)	*Prunus*	-	[95]
		ssRNA (+), ~9.6 kb		Clover yellow vein virus (CYVV)	*Fabaceae*	-	[96]
		ssRNA (+), ~9.5 kb		Tobacco etch virus (TEV)	*Solanaceae*	+	[97]
Potyvirus, Poacevirus	Filamentous monopartite	ssRNA (+), ~9.5 kb	Mites, mechanical	Triticum mosaic virus (TriMV)	12 species of grasses	-	[98]
Tombusvirus	Icosahedral monopartite	ssRNA (+), ~4.8 kb	Mechanical, soil	Tomato bushy stunt virus (TBSV)	Over 120 plant species across 20 families	+	[99]
